# Digital transformation of pathology - the European Society of Pathology expert opinion paper

**DOI:** 10.1007/s00428-025-04090-w

**Published:** 2025-03-31

**Authors:** Catarina Eloy, Filippo Fraggetta, Paul J. van Diest, António Polónia, Mónica Curado, Jordi Temprana-Salvador, Inti Zlobec, Elvira Purqueras, Cleo-Aron Weis, Xavier Matias-Guiu, Peter Schirmacher, Aleš Ryška

**Affiliations:** 1 Pathology Laboratory, Institute of Molecular Pathology and Immunology, University of Porto (IPATIMUP), Rua Júlio Amaral de Carvalho 45, 4200-135 Ipatimup, Portugal; 2 Pathology Department, Medical Faculty, University of Porto, Porto, Portugal; 3 Pathology Department, Gravina Hospital, Caltagirone, ASP Catania, Italy; 4 Department of Pathology, University Medical Center Utrecht, Utrecht, Netherlands; 5 Escola de Medicina E Ciências Biomédicas, Universidade Fernando Pessoa, Porto, Portugal; 6 Department of Pathological, Cytological and Thanatological Anatomy, School of Health of Polytechnic Institute of Porto, Porto, Portugal; 7 Department of Pathology, Vall Hebron University Hospital, Barcelona, Spain; 8 Institute for Tissue Medicine and Pathology (ITMP), University of Bern, Bern, Switzerland; 9 Hospital Universitari de Bellvitge, IDIBELL, Barcelona, Spain; 10 Institute of Pathology Heidelberg, Heidelberg University, Heidelberg, Germany; 11 Hospital U Arnau de Vilanova & University of Lleida IRBLLEIDA, Lleida, Spain; 12 The Fingerland Department of Pathology, University Hospital Hradec Králové & Charles University Medical Faculty, Hradec Králové, Czech Republic

**Keywords:** Digital pathology, Recommendations, Digital transformation

## Abstract

An expert group mandated by the European Society of Pathology (ESP) outlines its recommendations on the digital transformation of pathology departments, aiming to facilitate the acquisition of resources for better patient care. This statement is directed at pathology professionals, offering guidance for the safe implementation of digital pathology while emphasizing the necessity of standardization, quality control, and sustainability. Digital pathology involves automating and standardizing laboratory workflows to produce high-quality whole slide images (WSIs), which are crucial for diagnosis, research, and education. A successful digital transformation requires a multidisciplinary approach, significant investment in human, structural, and informatic resources, and progressive adaptation of laboratory workflows. Key components include robust infrastructure; continuous training; and clear policies for hardware renewal, data storage, and interoperability. The transition demands attention to quality and production control, ensuring efficient WSI generation and timely diagnostic reporting. ESP strongly recommends that pathology departments, supported by funding organizations, start to prioritize digital transformation as a step toward improved patient care and in alignment with global healthcare initiatives. Collaboration, investment, and adherence to quality standards are critical to benefiting the most the full potential of digital pathology.

## Aim and target of this statement

These recommendations represent the position of experts appointed by the European Society of Pathology (ESP), including pathologists, biomedical scientists, and laboratory technicians. These recommendations provide guidance to the safe implementation of digital pathology and smooth transition into a digital era. Considerations on computational pathology are out of the scope of this document, as are issues regarding the business case for digital transformation.

## Definitions and general concepts

Digital pathology (or digital transformation of pathology) is a process that entails the implementation of procedures at the pathology departments ultimately aiming to produce high-quality whole slide images (WSIs) for a diagnostic use. All processes must be maximally standardized to guarantee reproducibility and reliability of pathologic diagnosis. The concept of standardization is well established in the traditional (analog) workflow; digital transformation requires even more robust and well-tailored quality control [1–3].

Implementation of digital pathology workflow brings certain immediate benefits, such as better workflow efficiency. It enables remote access to cases, when pathologists can review slides from anywhere, enabling work-from-home and facilitating consultations. Simultaneous viewing allows in selected cases multiple pathologists examine the same slide concurrently. A critical moment is the improved case organization, as digital slides are organized systematically and cannot be lost, misplaced, or broken. It has been repeatedly reported that digital transformation helps faster case review due to quick navigation between slides and cases without physical handling. Another benefit is the better workload distribution: reduced administrative burden thanks to automated case assignments and tracking as well as prioritization of critical or urgent cases.

Also, the impact on improvement of diagnostic quality with more precise measurement tools for quantification of quantitative features (tumor size, distance from margins, measurement of area, or percentage of involved tissue). Use of virtual slides brings the possibility of side-by-side comparison (two or more slides with different staining, comparison with previous specimens or reference images). Use of WSIs is the prerequisite for image analysis as well as use artificial intelligence (AI) algorithms [4, 5].

Long-term benefits include better research opportunities with easier access to large case cohorts for studies, which are immediately available and a need for additional physical slides and associated extra costs for reagents and manpower is thus reduced.

The digital workflow in the laboratory can be better connected with other systems and brings straightforward integration with hospital information systems and electronic health records.

This is also the path to enhancement of collaboration and use in education. It facilitates intra- and interdepartmental consultations, can be used for multidisciplinary team meetings, creation of teaching collections, and slide libraries for education, resulting in more standardized training using consistent educational materials.

Benefits can be potentiated at the regional or national level by support of multicentric structured digital, e.g., by the governmental institutions [6–9].

The digital transformation of pathology should represent a natural evolution of pathology as a diagnostic discipline resulting in improvement in healthcare assistance with positive consequences on the management of patients. Partially, digital workflows bring only limited benefits as compared to fully digital workflow. The partial implementation of digital pathology (hybrid workflow) can be considered as an intermediate step on the path to fully digital status. However, coexistence of digital and analog workflow may create important challenges in the daily routine as it may impact quality of pathologic diagnosis (see discussion below) [10]. The digital practice offers potential for collaboration with industry partners, namely in the setting of clinical trials, stimulating further development and continuous improvement of technology [10]. In addition, digital transformation may also constitute a powerful factor in educating and attracting young generations of pathologists and laboratory technicians/medical scientists, and thus contribute to overcoming the shortage of human resources in pathology [11]. The principles of digital pathology should be included in the curriculum of pathology specialization, and specific educational measures on the subject are warranted [12].

For all the abovementioned reasons, it is highly recommended to accelerate the digital transformation of pathology, coping also with the general trend in other healthcare services [13].

## Management of resources

**Fig. 1:**
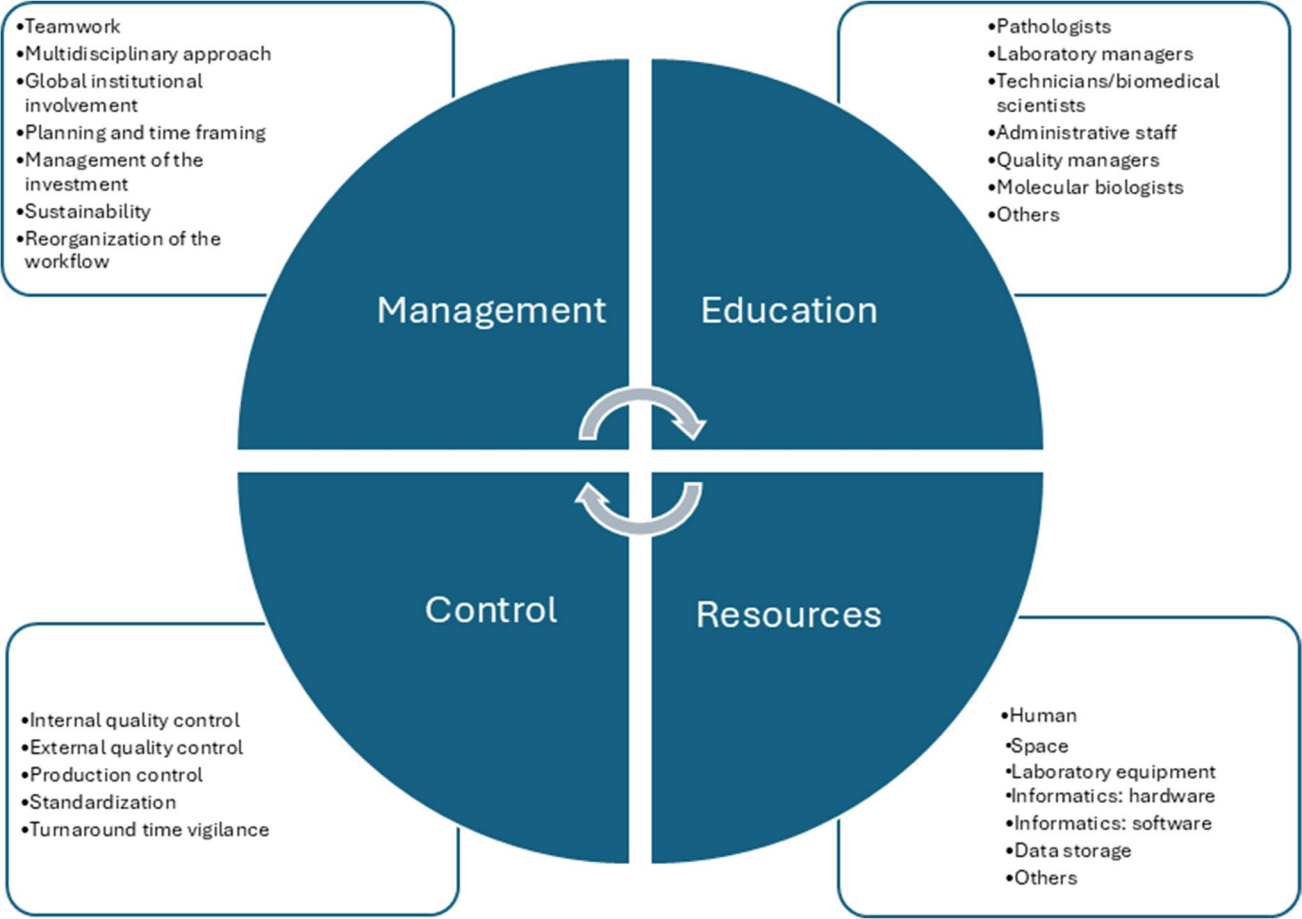
Management, education, resources, and control policies involved in the digital transformation of pathology

The digital transformation requires a significant investment in the management of human, structural, and dedicated informatic resources [9, 14, 15]. Such investment does represent not only financial resources, but also educational and structural/organizational changes (Fig. 1). Their extent is directly dependent on the baseline status of the department. It should be conducted progressively, according to a pre-established plan. It is very important to keep in mind that investment is not limited to the initial purchase of digital technologies (hardware, software) and change of the processes in the department. It represents an ongoing commitment required to keep all processes up to date, reflecting the needs of developing technology, which may become outdated even faster than in traditional (analog) environment. Renewal of digital technology equipment is more frequent but at lower costs than the initial investment, unlike with the analog workflow renovations. In particular, the need for additional storage capacities requires permanent investment. Fortunately, as the storage capacity cost is decreasing in time, this represents progressively declining burden for the departmental budget [16, 17].

### Human resources

 The successful digital transformation of the department requires expert-driven leadership to coordinate operations and staff. This responsibility is best taken on by a qualified pathologist with the necessary expertise and institutional mandate, who will constitute and work closely with a multidisciplinary team to plan and implement each step of the process in due time. The multidisciplinary team should encompass pathologists, laboratory technicians/medical scientists, computational scientists/informaticians, the laboratory quality manager, administrative staff, and all other personnel considered adequate to contribute [2, 3]. It is a key step to inform the full department staff about the goals, timeframe, and planned tasks of the digital transformation.

Human resources management also represents the identification of specific educational needs guaranteeing adequate knowledge in the field or incorporate new staff with dedicated skills. Some tasks become obsolete, and others enter the scenario, mainly for laboratory technicians. Targeted meetings at each of the workstations, either from technical staff or from medical staff, are required to identify individual issues and potential bottlenecks [8, 18]. The administrative, technical, and medical staff need to be educated in this subject and engage in training opportunities to facilitate conceptual acceptance and to accelerate the implementation.

### Structural/laboratory resources and technical challenges

The leading pathologist, together with the selected team, shall identify structural requirements needed, namely additional space for scanning equipment, change of the sample path within the building/rooms, reorganizing the laboratory and processes in time, identifying and selecting instruments allowing digital navigation, automation of stable production of glass slides, and subsequent WSIs for the diagnosis [2].

The implementation of digital pathology requires investment in equipment—both directly related to the manipulation of WSIs (scanners, computers, monitors, servers) and laboratory equipment related to quality of glass slides (microtomes, stainers, coverslippers). Need for large investment may be reduced by implementation of continuous workflows and reorganization of the laboratory procedures.

The quality of glass slides severely impacts the quality of WSIs [19], and the suboptimal quality of histological sections is one of the major reasons for the lack of sustainability of the digital workflow [16]. Sample management from accessioning until the release of the report must be traceable and audited [2, 8]. Due to the bottleneck effect at the scanning workstation(s), scanning of the slides adds time to the total turnaround time of each sample. It is, therefore, necessary to take simple measures to assure maximum quality of glass slides prior to scanning [20–22]. This eliminates the need for repetition of preparation of the slides and allows very high levels of *first time-right* sample management. The major consequence of this good practice is very low rate of rescans, which should be systematically recorded and kept below 1%. Rescan value may serve as one of the quality indicators and thus represent also a measure of the sustainability of production.

The preparation of tissues at the gross examination requires respecting the maximum thickness of the tissue in the cassette to ensure adequate tissue processing and maintaining the tissue dimensions to not exceed the scanning area. Inking of low-contrast/hypocellular samples such as cellblocks; lipomatous tumors; and other fatty tissues, such as breast biopsies, helps better identification of the tissue by automated algorithms of the scanner software [21]. At the embedding workstation, fragments should be gathered as close as possible to each other, not overlapping [8]. Microtomes should be calibrated more frequently allowing very thin, homogeneous, and flat sections (ideally 2–3 µm) with minimal artifacts (tears, bubbles, folds, precipitates) [19, 22]. Sections must be placed in the center of the slide allowing full coverslipping. If multiple sections are placed on the same glass slide, non-scanned areas specific for each scanner brand/scanner model should be avoided. The selection of the type of slides used for haematoxylin–eosin (HE) staining may impact the turnaround time since scanning of the sections placed on adhesive slides takes more than double time and results in larger file size due to scanning of stained background [22]. Independently of the type of coverslipping, slides must be fully dried and cleaned before loading to scanner racks, to prevent poor-quality scans, misplacing of the coverslip, and potential damage of the scanner. The coverslipping method interferes with the quality of the WSIs, scanning time and file size, so automated coverslipping and scanner calibration is recommended [20].

The quality of scanning process can be controlled by the comparison of the WSI with the respective macroscopy image of the tissue block, the glass slide, or the scan preview image (to confirm that all fragments were scanned, to check presence of out-of-focus areas, stitching artifacts, others). Each department should establish a quality policy regarding the verification of scanned slides, either all slides or just specifically selected or random sample. During this verification, evaluation of all previous laboratory steps should be included; this can be performed by an operator or by a dedicated software [23]. After scanning, glass slides are ready to be archived and WSIs are immediately available to the pathologist for reporting through laboratory information system (LIS/LIMS) and/or picture archiving and communication system (PACS). Of note, case assignment in the digital workflow (including prioritization) can be done automatically by the LIS/LIMS, accelerating this task [24].

Although WSIs should be a complete and precise digital equivalent of glass slides, sometimes not all tissue fragments are automatically identified and scanned. As such, pathologists must always have an easy access to the scan preview image, which represents a low-resolution snapshot of the entire glass slide, used to compare with the WSI and make sure that every tissue fragment is scanned. This may be also performed automatically by a dedicated software [25].

Scanning special stains and immunohistochemistry (IHC) slides may require another level of technical interventions.

Most scanners digitize IHC samples without any issues. Even though it is not as complex as digitizing cytology or fluorescence slides, it has still some differences from HE. Mainly, hematoxylin counterstain can be very pale, and thus some tissue detection algorithms can fail to detect the whole sample. The same issue can happen with histochemistry staining (especially weak green or blue stains). It might be helpful to highlight some small samples with ink to ensure tissue detection in all cases, as stated above, or to adapt the scanning protocols, e.g., by increasing the number of focus points or resolution of the image. In some cases, control tissue on the same slide can affect tissue detection for the scanner. If the sample is extremely small, it may be ignored by the scanner. Too large sections may prevent placement of control tissue within the limits of scanning area. Also, control tissues increase the file size and scanning time. Still, for robust quality control, it is recommended to always include control tissue in the WSI. IHC WSI are essential for quantification of diagnostic biomarkers using digital algorithms [26, 27].

Cytology slides are among the most difficult slides to scan [28]. Compared to histology, the implementation of digital cytology drop behind [16, 29]. This is caused by the fact that cytology specimens (even monolayers) usually have a greater third dimension (*z*, thickness) than histology sections. Single-plane scans of cytology slides may suffice for demonstration and teaching purposes but do not allow proper assessment of cell groups and even individual cells for diagnostic purposes. Some high-throughput scanners (primarily developed for scanning histology slides) are able to scan smears in multiple focus planes, however at the cost of longer scanning and viewing times as well as larger file size. Some digital cytology validation studies have been published showing acceptable performance [30, 31], but few applications only have reached more widespread clinical use [29]. Fortunately, a new generation of scanners is now entering the market, where the slide (cytology or histology) is scanned in one flow (“volumetric” scanning) with acceptable scanning time, creating an artificial single plane WSI of standard file size. Recently, an FDA approval has been obtained for an AI algorithm used to select and display the representative cells from volumetrically scanned cervical thin layer slides for fast review [32]. In view of the limited experience with digital cytology, thorough clinical validation is recommended irrespective of the digital cytology platform used [33, 34].

### Informatic resources: hardware requirements including diagnosis-dedicated workstations

 Key hardware elements are servers to run the PACS and/or LIS/LIMS, network connections, scanners, storage facilities, as well as digital workspace of pathologists. The servers running the PACS and/or LIS/LIMS must have an optimized up-time since the complete workflow efficiency depends on this. Network connections must have sufficient bandwidth to swap the large WSIs between scanners, storage servers, and workspace of the pathologists. Of those, the bandwidth between scanners and storage servers is most critical (preferably 1000 Mbit), while for streaming WSIs to workspace computers, lower bandwidth (10 Mbit or even good Wi-Fi) will suffice.

Slide scanners must meet the specific needs and reflect the department workload. Scanning at 40 × or the equivalent magnification is considered standard today (0.25 µm/pixel); although, some scanners use even higher magnifications, useful for specific cases or stains. Scanner features to consider are (besides price): scanning speed, single or multiple focus scanning, ability to scan also macro-slides, dark field slides, and cytology slides. Other features to ensure speed, reliability, and to avoid jamming, like automatic rescan and image quality assessment, are helpful. Scanning software should allow multiple profiles to be created (e.g., HE, cytology, IHC), adjusting focus points and automated quality control/rescan options. Also, the output file format must be compatible with various PACS and/or LIS/LIMS systems and preferably provide DICOM output or at least allow conversion without significant delay and increase in file size. Scanners produce heat, noise, and ozone, so the option of a separate climate-controlled scanner room should be considered. Still, it is advantageous to put the scanners in proximity to production line sites at the laboratory. When deciding on a scanning solution, it is important to consider scanner deployment, including contingency, workflow, and timing, as well as adaptation to functional needs. In this context, for example, the size of the device or the possibility of stacking the scanners could be factors considered in the light of limited laboratory space.

Storage capacity of the digital archive must be tuned to the needs considering temporary versus permanent storage, on-site versus remote (even cloud) storage, access time (determining the total storage price), sustainability, and CO_2_ footprint. The permanent storage of images is essential for medico-legal proof of the basic substrate for diagnosis (since image manipulation is not acceptable in any circumstance), and accelerates slides retrieval for additional review, research (namely on AI), and education.

The workstation of the pathologist, in contrast to the optical microscope, requires hardware for the visualization of WSIs—a computer with computational capacity, computer screens, and a navigation tool. As a rule of thumb, 16.00 GB of RAM is considered a minimum for routine work, preferably scalable to at least 32.00 GB of RAM. An appropriate GPU is needed for image analysis, namely if AI use is planned. The development of hardware is extremely rapid. Therefore, no specific recommendations regarding minimal requirements for computer workstations are given here, as they would become very soon obsolete.

For image display, it is recommended to have at least two computer screens, one for image-viewing and another one for non-image-viewing display. It is important to be aware that the technical specifications of the display, such as size, resolution, luminance, and contrast interfere with the quality of the image where the medical assessment, is to be taken into consideration. The minimum specifications of the image-viewing display suited for digital navigation in pathology are debatable. Routine diagnosis may require medical-grade monitors, but the commercial-grade ones may also be appropriate for digital pathology use. Non-image-viewing monitors can be commercial-grade displays to support applications such as LIS/LIMS and the electronic health record. Screen size is a relevant factor when observing WSIs. Larger screens are usually preferable, with diagonal sizes between 24 and 32 in being the most used. The most appropriate diagonal size is three-fourths of the viewing distance. Regarding screen resolution, full high definition (FHD, 1920×1080 pixels) is considered a minimum, but higher resolution of the screen provides clearer image not being influenced by pixel visibility. Additionally, it has been recommended that a maximum luminance of at least 350 cd/m^2^ [35]. While color calibration is not a critical factor when making a qualitative assessment or diagnostic accuracy, it significantly impacts interpretation speed and comfort of the pathologist [36]. However, if in the future quantitative color analysis becomes more relevant, color calibration at the staining, scanning, and viewing steps will become mandatory [37]. Currently, there is no accepted standard prescribing how displays need to be color calibrated [38]. Importantly, when using digital measurements, resolution calibration is essential to make sure that scanned images are captured to scale [39]. One strategy to validate the entire diagnostic setting, including the viewer display, the pathologist visual system, and the local environment, is to use the freely available web-based application point-of-use quality assurance tool for digital pathology (https://www.virtualpathology.leeds.ac.uk/research/systems/pouqa/pathology/) [40].

Poor-quality imaging displays can result in misdiagnosis, eye fatigue, and headache. Additionally, some modifications of the local environment may be necessary to optimize the observation of the samples, such as low ambient lighting and control of display reflections. It has been shown that the performance of color evaluation is best with artificial light rather than natural light. Displays should never be placed in direct sunlight, and the use of a black-out blind can help prevent the presence of excess natural light entering the room [40]. FDA recommends adhering to a closed “pixel pathway” (encompassing all equipment from scanner to display) and any modification of the system is considered an off-label issue, requiring validation as a laboratory developed test (LDT) [41].

Lastly, navigation tools to easily evaluate the WSIs are a matter of personal preference and ergonomics [42]. A large variety of instruments exist on the market with different price ranges. The regular mouse is the most popular choice. A vertical mouse usually maintains the hand in a more ergonomic position with the forearm, without inward rotation of the forearm and preventing unnecessary stress to the wrist joint. The use of a mouse pad with wrist rest also supports the wrists using the mouse, reducing discomfort with prolonged use.

The number of available microscopes may be reduced, as not every pathologist may need a private microscope anymore, but some must still be available (e.g., outside slides, cytology, poor-quality WSIs).

### Informatic resources: software connectivity, interoperability, and traceability challenges

One of the most important steps in planning the digital workflow is designing software architecture. Proper uni-or bilateral connections will be required between the various components (scanners, PACS, LIS/LIMS, storage, hospital information system, billing engines, speech recognition software, synoptic reporting, centralized archives, consultation servers, image analysis/AI servers) to make sure the right information is available at the right moment all the time. The flow of information must comply with a recognized standard such as Health Level Seven International (HL7), including FHIR (Fast Healthcare Interoperability Resources), to provide a comprehensive framework and standards for the exchange, integration, sharing, and retrieval of electronic health information [43]. This digital framework will support clinical practice and the management, delivery, and evaluation of healthcare services.

The PACS must allow workflow design, viewing WSIs including tracking of viewing, making annotations and measurements, consulting and chat with colleagues, evaluation of WSIs side-by-side, importing images from other sources (request forms, grossing images, electronic microscopy, and autopsy pictures), and exporting images. The PACS should guarantee interoperability, be able to accommodate various proprietary scanner and DICOM file formats, and allow integration of image analysis and AI plugins.

A LIS/LIMS centered approach supported with requests, files, and software (including storage) and equipment (tissue processors, slide stainers, scanners) is essential in all clinical settings to assure interoperability. Photographic (cameras), voice or video, systems can be implemented in several laboratory steps. The LIS/LIMS is also the central element of the specimen tracking system (including bidimensional code printing and reading).

An adequate specimen tracking system is a prerequisite for the introduction of a digital workflow [2].

Unique bar codes or similar systems are essential for sample traceability, i.e., the ability to track the sample through the different processing stages from accessioning to reporting. 2D barcodes (QR codes) are more versatile than 1D barcodes as they take up less space, fit well on the tiny surface of both tissue blocks and glass slides; are easier to apply to the convex surfaces of tissue containers; and due to redundance of the encoded information are generally less prone to scanning problems. The same tracing code is placed on the request form, sample container, printed on the cassettes, slides (as adhesive labels or directly on the glass), and on the report. All workstations must be equipped with code readers, and some workstations include code printers (cassette printers, slide printers). This guarantees a chain of custody from accepted samples to the respective WSI.

WSIs can be viewed or exchanged in the framework of so-called telepathology. Telepathology deals with the transmission and remote interpretation of pathological (macroscopic and microscopic) images of tissue or cell samples [44–46]. This allows us to improve the quality and speed of diagnoses, facilitates collaboration, and reduces costs and reporting times. Telepathology includes teleconsultation (requesting a second opinion), teleeducation (use of pathological images for educational purposes), teleconference (discussion of clinical cases among the members of multidisciplinary team or tumor board under the pathologist’s supervision), and remote diagnosis (without the need to send the glass slide). The clinical use of a telepathology model, namely for remote diagnosis, requires the maintenance of interoperability and safety to ensure the protection of personal data and the confidentiality of health information, in compliance with national and international regulations. The access to pathology WSIs should be restricted to pathologists to prevent their misinterpretation by users without appropriate pathology training.

The pathology report is the prototype of the electronic record that constitutes the most relevant source of data. The possibility to use dedicated LIS/LIMS adapted to digital workflow allows the evolution from a narrative reporting to a synoptic reporting system by standardizing data elements to ensure consistency, structured formats that improve completeness and scalability. Synoptic reporting also generates structured data that can be used for research or studied by other disciplines such as epidemiology, bringing additional benefit of histopathological reports [43, 47].

## Control measures

### Quality control

Independently of choosing to undergo digital transformation or not, each department must have implemented various measures of internal and external quality control to warrant the quality of the diagnosis provided to the clinical users (and ultimately to patients). Quality control measures must comply with local/national and international regulations. Mentioning the digital workflow in the diagnostic report of the case is advisable and is included as a requirement in some accreditation systems.

Several international recommendations, position papers, or guidelines have been published on the topic of validation of digital observation for diagnostic purposes, and some are mentioned here. While some recommendations refer to a global evaluation of the entire diagnostic chain as whole, others suggest the need to validate each instrument and piece of the workflow individually. Each department should choose the recommendation/guideline that is best fitting for the purpose of their practice.

The College of American Pathologists (CAP), in collaboration with the Laboratory Quality Center (LQC), published their original guideline in 2013 to answer the fundamental question “What needs to be done to validate a WSI system for diagnostic purposes before it is placed into clinical service?” [48]. This paper contains 12 guideline statements. Later, in 2021, CAP, in collaboration with the American Society for Clinical Pathology (ASCP) and the Association for Pathology Informatics (API), published an updated version of the 2013 guideline [49]. This paper contains three strong recommendations and nine good practice statements. The use of a validation set of at least 60 cases reflecting the spectrum and complexity of specimen types and diagnoses likely to be encountered during routine practice is recommended establishing interobserver diagnostic concordance between WSIs and glass slides to closely emulate the real-world clinical environment in which the technology will be used. The validation process should include another 20 cases to cover IHC or other special stains if these were not included in the initial 60 cases. A washout period of at least 2 weeks should occur between viewing digital and glass slides. Diagnostic concordance between digital and glass slides of at least 95% should be reached for the same observer. This validation design does not preclude additional validation procedures or an extra number of cases to validate specific settings such as frozen sections or dark field observations. The validation study should encompass the entire system, i.e., it is not necessary to separately validate each individual component of the system or the individual steps of the digital imaging process, making it very practical. The range of the recommendations towards cytological specimens has expanded recently in a dedicated publication [50].

One of the first national documents published in Europe to regulate the use of WSI was the “Best Practice Recommendations for Implementing Digital Pathology” by Cross et al. on behalf of the Royal College of Pathologists. It offers recommendations based on existing evidence and practical experience [51]. It included definitions, guidance for validation and quality control, regulation compliance, and special considerations on remote diagnosis.

Also in 2018, the Professional Association of German Pathologists published the “Digital Pathology in diagnostics-reporting on digital images” [52]. The document outlines guidelines for implementing digital pathology (DP) systems, particularly virtual microscopy using WSI. Key topics include system validation, requirements for slide scanners, image quality control, and integrating scanners with pathology information systems. The guidelines emphasize maintaining diagnostic accuracy equivalent to conventional microscopy, ensuring data security, and compliance with international rules. It also covers various technical components, such as image compression, archiving, and monitor specifications, aiming to standardize and secure long-term investments in digital pathology solutions.

Afterwards, the European Society of Digital and Integrative Pathology (ESDIP) provided consensus-based recommendations, which were developed through expert discussions [2]. This document offers a comprehensive practical reference for adopting digital workflow, focusing on interoperability, automation, and tracking processes.

More recently, the Swiss recommendations for digital pathology [53] are based on the Delphi approach and are divided into four main topics:

1. Scanners, quality assurance, and validation of scans.
2. Integration of WSI-scanners and digital pathology systems into the LIS.
3. Quality requirements for laboratory staff and technicians, as well as for the digital workflow for pathologists.
4. Image analysis (IA)/ AI. The results are 83 consensus statements.

The above cited guidelines refer to histopathology, and at this stage limited experience is available regarding the use of WSIs in cytology that have been recently approached by the American Society of Cytopathology Digital Cytology Task Force [33, 34].

From all the abovementioned sources, it was made clear that the digital transformation requires reinforced internal quality control and opens the need for dedicated external quality control programs tailored to aim the digital observation. Several external quality assessment (EQA) providers today offer their EQA programs using WSIs [54–57], usually referred to as “virtual microscopy,” sometimes claiming to provide EQA in digital pathology. However, this would be a significant misinterpretation of the meaning of EQA in digital pathology. While EQA programs for diagnostic histopathology and clinical cytology based on virtual microscopy can undoubtedly benefit from digital pathology technology to share EQA cases globally and provide identical images to a virtually unlimited number of participants, the only steps of the diagnostic workflow controlled by such a program are the projection (screen quality and settings) and slide interpretation by the pathologist. All processes preceding this step, such as slide preparation, scanning, and data compression, are not covered. There is no doubt that successful participation in the EQA program(s) using WSIs is important and demonstrates the proficiency of the participating pathologists in the areas covered by the program. However, a much more complex EQA scheme covering the remaining parts of the digital workflow will be needed to demonstrate the quality of all individual steps of the digital process. A program promising to provide such a complex EQA has been recently announced (Digital Pathology (PILOT), https://www.ukneqascpt.org/DigitalPathology), but is not available at the time of publication of this paper.

### Production control

 The process of scanning introduces a new bottleneck element in the workflow since scanners usually scan one slide at a time. An adequate number of scanners adjusted to the expected daily workload helps to minimize the delay caused by scanning. The compromise between the ideal number of scanners and the financial impact of the purchase of the instruments as well as the associated resources availability may be delicate and should be managed accordingly by each department. However, there are also other measures to accelerate scanning without dramatically increasing the cost of the whole process.

The most important one is the adoption of continuous glass slide production, which eliminates bottlenecks in the laboratory. As a result, the slides are more evenly distributed during the day, and this results in smaller batches of slides at different stages of tissue processing. Continuous workflows lead to less accumulation and shorter waiting time at the scanner station, thus increasing speed and efficiency of the entire process.

Keeping the hybrid workflow with glass slides used in parallel with WSIs (scanning of just a percentage of selected cases) multiplies the procedures, creates parallel workflow tracks, and decreases efficiency, jeopardizing sustainability of digital transformation. Another consequence of the hybrid workflows is interference of the two tracks (analog and digital) at certain points, requiring different validation procedures. Moreover, the department can neither fully benefit from digital archive (immediate access to previous slides) nor widely use computational tools in the management of the workflow.

The duration of each step in the slide preparation process, such as the time taken for embedding, microtomy, staining, and slide quality control, as well as the waiting time between individual tasks and the time needed to repeat poor-quality operations, should be recorded, closely monitored and regularly reviewed. It is recommended that production control tools, such as the lean system, be incorporated into the department information system. Such steps can help to minimize the total slide preparation turnaround time (total time between receipt of the specimen and availability of the corresponding WSIs to the pathologist). A highly efficient workflow with rapid reporting eliminates the need to implement special fast tracks for urgent samples, development of any irregular shortcuts, as well as preliminary reporting.

All pathologists are aware that the value of a pathology report lies not only in its extent, accuracy, and completeness, but also in the timely delivery of the result; a value that can be amplified by digital transformation.

## Conclusions

It is the sincere belief of the authors that digital pathology is the future of our discipline and digital workflow will be implemented in our routine practice. Therefore, digital transformation of pathology should be seen by the pathology departments as a priority goal, which can be achieved independent of size and setting of the department, supported by adequate funding, to cope with the best standards for patient care.

For the safe and sustainable digital transformation process, adequate investment in instruments, informatic resources, and their implementation, as well as reorganizing the structure of the laboratory are needed and must be recognized by all partners to guarantee the best practice.

The consistent framing of the digital transformation by control measures should be supported by the well-established means of quality control, both internal and external, incorporating regular validation procedures according to existing regulations, as well as a production control to support efficient and timely reporting.

## Data Availability

Not applicable.
